# Inferring high-confidence human protein-protein interactions

**DOI:** 10.1186/1471-2105-13-79

**Published:** 2012-05-04

**Authors:** Xueping Yu, Anders Wallqvist, Jaques Reifman

**Affiliations:** 1 Biotechnology High-Performance Computing Software Applications Institute, Telemedicine and Advanced Technology Research Center, U.S. Army Medical Research and Materiel Command, Ft. Detrick, MD, 21702, USA

**Keywords:** High confidence, Human protein interaction network, Protein-protein interactions

## Abstract

**Background:**

As numerous experimental factors drive the acquisition, identification, and interpretation of protein-protein interactions (PPIs), aggregated assemblies of human PPI data invariably contain experiment-dependent noise. Ascertaining the reliability of PPIs collected from these diverse studies and scoring them to infer high-confidence networks is a non-trivial task. Moreover, a large number of PPIs share the same number of reported occurrences, making it impossible to distinguish the reliability of these PPIs and rank-order them. For example, for the data analyzed here, we found that the majority (>83%) of currently available human PPIs have been reported only once.

**Results:**

In this work, we proposed an unsupervised statistical approach to score a set of diverse, experimentally identified PPIs from nine primary databases to create subsets of high-confidence human PPI networks. We evaluated this ranking method by comparing it with other methods and assessing their ability to retrieve protein associations from a number of diverse and independent reference sets. These reference sets contain known biological data that are either directly or indirectly linked to interactions between proteins. We quantified the average effect of using ranked protein interaction data to retrieve this information and showed that, when compared to randomly ranked interaction data sets, the proposed method created a larger enrichment (~134%) than either ranking based on the hypergeometric test (~109%) or occurrence ranking (~46%).

**Conclusions:**

From our evaluations, it was clear that ranked interactions were always of value because higher-ranked PPIs had a higher likelihood of retrieving high-confidence experimental data. Reducing the noise inherent in aggregated experimental PPIs via our ranking scheme further increased the accuracy and enrichment of PPIs derived from a number of biologically relevant data sets. These results suggest that using our high-confidence protein interactions at different levels of confidence will help clarify the topological and biological properties associated with human protein networks.

## Background

The development of high-throughput techniques during the last decade has led to an unprecedented increase in the volume of identified human protein-protein interactions (PPIs). The currently available individual PPI data sets can be roughly categorized into three sets: *1*) proteome-wide, large-scale screenings aimed at investigating all possible PPIs [1-3], *2*) semi-large-scale screenings aimed at investigating the interactions between a specific group of proteins (typically in a pathway) and all other proteins [4,5], and *3*) small-scale, traditional studies aimed at detecting specific PPIs among biologically interesting proteins, e.g., oncogenes and their regulators. Although this latter set is still numerically dominant (~80% of all PPIs belong to this set), examples of the first two types of investigations are expanding rapidly.

Given this extensive resource of known human PPIs and their continuous accelerated growth, how to globally analyze and aggregate the data remain a challenge. Statistical methods for inferring confidence of protein interactions can be broadly divided into two groups [6-8]: scoring schemes that rely on the interaction data themselves (e.g., affinity purification/mass-spectrometry [AP/MS] data or yeast two-hybrid [Y2H] data) and scoring schemes that require additional data sources not directly related to the interactions *per se* (e.g., functional annotation or gene expression data). Herein, we address the question of how to extract high-confidence PPIs while relying only on the aggregated interaction data themselves.

The most intuitive approach to infer high-confidence PPIs is to score PPIs based on the number of times an interaction has been reported [9-11]. However, using the number of times a PPI has been reported (occurrence) across different studies as the metric of reliability could be influenced by numerous unknowable experimental factors, e.g., recent studies have demonstrated that such factors may result in decreased reliability of PPIs containing frequently studied proteins [12]. Moreover, a large number of PPIs share the same number of reported occurrences, making it impossible to use occurrence alone to establish the reliability of these PPIs and rank-order them. For example, for the data analyzed here, we found that the majority (>83%) of currently available human PPIs have been reported only once.

Herein, we propose an unsupervised statistical approach to score and rank a set of diverse, experimentally identified PPIs. We applied this methodology to human PPIs (non-physical associations excluded) aggregated from nine publicly available primary databases that exclusively contain experimental data (Additional file 1): the Biomolecular Interaction Network Database (BIND) [13], the Biological General Repository for Interaction Datasets (BioGRID) [14], the Database of Interacting Proteins (DIP) [15], the Human Protein Reference Database (HPRD) [16], IntAct [17], the Molecular INTeraction database (MINT) [18], the mammalian PPI database of the Munich Information Center on Protein Sequences (MIPS) [19], PDZBase (a PPI database for PDZ-domains) [20], and Reactome [21]. Our method re-normalizes the importance of frequently occurring proteins among PPIs to avoid giving added (and potentially artificial) weight to those interactions. We estimated the importance of a PPI by comparing the actual observed occurrence of a PPI with its occurrence in a randomized sample. This calculation gauges the likelihood that the interaction occurs by chance in the set of all observed PPIs. Using these estimates, we rank-ordered the aggregated input PPI data set, allowing us to create high-confidence subsets based on a given rank threshold. At the lowest ranked threshold, all interactions are included and there is no difference between the ranked data and the original set of PPIs.

The presented scoring and ranking procedure can be seen as an extension of our previous effort to infer high-confidence interactions from the affinity purification raw data, termed interaction detection based on shuffling (IDBOS) [22], and, in the following, we will also refer to our scoring and ranking scheme as IDBOS. Our proposed procedure shares similarities to estimating probabilities of observed interactions above a random background based on the hypergeometric distribution [23], with the distinction that the IDBOS-generated probability density distribution functions correct for biases toward self-interaction among frequently studied proteins. Although other methods exist for assigning confidence scores to PPIs, these generally require additional data or reference sets [24,25], or *a priori* assumptions of network topology [26]. To the best of our knowledge, this is the first application of an unsupervised probabilistic scoring and ranking scheme to create subsets of unbiased high-confidence human PPI networks.

We evaluated the improvement in using IDBOS-ranked PPI data by comparing it with other methods and assessing their ability to retrieve biological associations from a number of diverse and independent reference sets. These reference sets contain known biological data that are either directly (e.g., crystallographically determined protein complexes) or indirectly (e.g., co-expressed genes) linked to interactions between proteins. The hypothesis we tested was that sets of highly ranked PPIs are enriched in biological associations as determined from the diverse reference sets. We quantified the average effect of using ranked protein interaction data to retrieve this information and showed that, when compared to randomly ranked interaction data sets, IDBOS created a larger enrichment (~134%) than either ranking based on the hypergeometric test (~109%) or occurrence ranking (~46%).

From our evaluations, it was clear that ranked interactions were always of value because higher-ranked PPIs had a higher likelihood of retrieving biologically relevant data. Statistically removing the biasing factors inherent in aggregated PPI data via the IDBOS-ranking scheme further increased the accuracy and enrichment of biological information associated with PPIs.

## Results

### Statistical scoring of human PPIs from literature data

We used the collection of human experimental and physical PPIs to create a set of 116,134 reported interactions, containing 80,980 unique physical associations between 13,369 distinct proteins (see Materials and methods). Out of the unique PPIs, 13,554 interactions were observed in more than one experiment. The number of times a PPI has been reported in the literature is an important metric for inferring high-confidence interactions [9,10,27], but it could also be dependent on other factors [12,28]. The observed number of interactions of a protein is partly reflective of how often it has been studied (popularity). For example, the top five connected proteins in the PPI data are G-protein beta subunit (GNB1), G-protein gamma subunit (GNGT1), G-protein alpha subunit (GNAL), ubiquitin C (UBC), and tumor protein p53 (TP53), having 2,280, 2,248, 2,243, 1,899, and 1,097 reported interactions, respectively. To normalize this popularity bias, we compared the observed number of protein interactions with statistics derived from the corresponding probability density distribution functions generated from randomized data. In the generation of the random interaction sets, we kept each protein’s reported number of interactions fixed and, thus, we expect the corresponding interaction probabilities of proteins with a high (low) number of reported interactions also to be high (low).

Note that the reported number of interactions involving a protein refers to the total number of observed PPIs in the literature involving that specific protein. This is different from a protein’s degree, which is defined as the number of unique interacting protein partners. Thus, while TP53 is associated with 1,097 observed PPIs, its degree is reduced to 478 due to the multiple observations of many of the involved interactions.

Our calculations followed our previously described procedures for generating Z-scores from sets of interacting protein pairs [22]. Briefly, the aggregated PPI data tabulates all pairs and the number of their occurrence in the literature. From this list we counted, for each unique pair between proteins *i* and *j*, how many times it occurred *O*_*ij*_. Randomized versions of the original PPI list were then created under the conditions that: *1*) the protein identifiers and the number of times they occur are preserved and *2*) no interactions are allowed between proteins of the same identifier, i.e., self-interactions are not allowed. We generated *M* = 10^6^ randomized PPI lists and calculated the average number of times each PPI from the original list occurred < *R*_*ij*_ > and its standard deviation σ_*R*_. The corresponding Z-score was then calculated as,

(1) $$Z_{\mathrm{ij}}=\frac{O_{\mathrm{ij}}-<R_{\mathrm{ij}}>}{\sigma_{R}}$$

We further used the randomized data to estimate the p-value p_*ij*_ of observing an interaction between proteins *i* and *j* in the original data set. Briefly, for each unique pair in the original PPI list, we calculated the number of times it occurred in each of the *n* = 1, … ,*M* random realizations and created the normalized probability density function PDF_*ij*_, i.e., the probability of finding $O_{\mathrm{ij}}^{R}=0\text{,}\;1\text{,}\;2\text{,}..\text{.}$ occurrences by random chance. We estimated the p-value for each actual interaction as,

(2) $$p_{\mathrm{ij}}\equiv \int_{\;O_{\mathrm{ij}}}^{\;\infty }PDF_{\mathrm{ij}}(x)dx\approx \frac{1}{M}\sum_{n=1}^{M}\{\begin{array}{c} \;1\;if\;O_{\mathrm{ij}}^{R}(n)\geq O_{\mathrm{ij}}\\ 0\;otherwise\; \end{array}$$

Z-scores and p-values (Z_*ij*_ and p_*ij*_) are legitimate metrics for ranking an observed PPI, although they are associated with different numerical properties and uncertainties because of the limitations imposed on using a finite number of randomizations.

### Assessing p-values and Z-scores as PPI metrics

**Figure 1:**
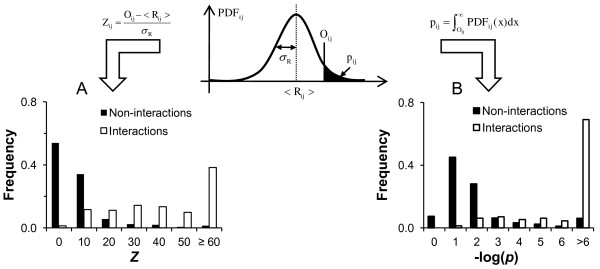
**IDBOS scoring schemes.** The method presented is an extension to the interaction detection based on shuffling (IDBOS) method used for mass spectrometry co-purification data [22]. We compared the set of known protein-protein interactions (PPIs) with randomized versions, which preserve the number of interactions per protein, to obtain a Z-score and a p-value for each interaction. These quantities are schematically outlined at the top, where a randomized probability density distribution function (PDF) is used to illustrate the p-value and Z-score calculations for a particular interaction between proteins *i* and *j*. To evaluate these scoring schemes, we analyzed interactions derived from crystallographic complexes in the PDB. Each human PPI was compared to a PPI derived from protein structure data in the PDB and assigned to one of two subsets: *interactions* or *non-interactions*. If the PPI was present in the PDB interaction data set, the pair was assigned to the *interactions* set, otherwise the pair was assigned to the *non-interactions* set. It is reasonable to assume that the first subset should be enriched with actual PPIs. (A) Distribution of Z-scores corresponding to “interactions” and “non-interactions” assigned to PDB-derived PPIs, and (B) the corresponding p-value distributions. We found that both p-values and Z-scores could distinguish these subsets, suggesting that they are useful metrics.

To assess the p-values and Z-scores as metrics in identifying and ranking high-confidence protein interactions in biological data sets, we compared the scored interactions with an independent PPI data set that was not part of the aggregated human data set. We used the human PPIs derived from experimentally determined structural protein complexes deposited in the Protein Data Bank (PDB) as outlined in the Materials and methods (Additional file 2). To assess our scored data using this data set as a benchmark, we selected only those interacting protein pairs from the scored human PPI data where both proteins appeared in the PDB-derived PPI data set. We termed these pairs as “judgeable,” i.e., the proteins appear in the benchmark data set, and we can judge whether the interaction occurred or not. Thus, for a given score, we determined how many of our judgeable PPIs were interacting and non-interacting within the benchmark set. Figure 1A-B shows the frequency of interacting and non-interacting pairs as a function of Z-scores and p-values. Interactions associated with high Z-scores and small p-values were enriched with benchmark interactions, indicating that both metrics were effective in extracting high-confidence PPIs from the aggregated literature data.

The procedure for shuffling the data allowed us to compare the frequency of each observed interaction *O*_*ij*_ to that interaction’s probability density distribution function PDF_*ij*_. This distribution is different for each interaction and depends on the number of distinct proteins and interactions present in the entire network. Lacking an analytical expression to generate the exact distribution functions, the procedure outlined in Equations 1 and 2 allowed us to generate estimates for both Z-scores and p-values for each interacting pair. We found that using either only p-values or only Z-scores to be inferior to using the combined information (data not shown), and, hence, we aggregated these two metrics by converting the value of each metric into a rank and generating the average rank from both metrics for each interaction. Interactions with the same p-value (or Z-score) were assigned the same rank. If interactions had the same rank in the final averaged-rank list, these interactions were considered equivalent and analyzed together. The ranked data are provided in the Supplementary materials (Additional file 3). Instead of assigning post-priori probabilities to already observed interactions, we only used ranks and comparisons between ranked data to gauge the biological information contained in these subsets.

### Top-ranked IDBOS PPIs are different from the most frequently reported PPIs

Table 1 shows the 20 top-ranked PPIs that were identified using the IDBOS system (sorted by average rank), and Table 2 shows the most frequently reported PPIs (sorted by PPI occurrence). As expected, the most frequently reported PPIs involved ubiquitously studied proteins, such as those mentioned above (UBC and TP53) as well as growth factor receptor bound 2 (GRB2), which had 916 reported interactions. These interactions, however, were not observed in the top PPIs scored by the IDBOS procedure. Instead, the top PPIs of the IDBOS set were enriched with infrequently observed interactions. Although we could not independently assess whether these interactions are more “real” than the frequently observed ones, they are partly supported by independent literature citations. In fact, the proteins in these interactions could be classified into two groups. The first group comprised proteins that had the same specific function or were subunits of the same protein complex, such as branched chain keto acid E1 alpha (BCKDHA) and branched chain keto acid E1 beta (BCKDHB) [3,29], and dynein cytoplasmic 2 intermediate chain 1 (D2LIC) and dynein cytoplasmic 2 heavy chain 1 (DNCH2) [30,31]. The second group comprised proteins that had a ligand/receptor relationship, such as inducible T cell co-stimulator (ICOS) and inducible T cell co-stimulator ligand (ICOSLG) [32-34], or gastric inhibitory polypeptide (GIP) and gastric inhibitory polypeptide receptor (GIPR) [35-38]. Among the top 20 PPIs of the IDBOS set, two seemingly unrelated protein pairs were actually closely related. The proteins in the LTC4S/MGST1 interacting pair are actually two of the six members of the membrane-associated proteins in the eicosanoid and glutathione metabolism (MAPEG) family. Similarly, the top-ranked interaction between L-threonine dehydrogenase (TDH) and aminoacetone synthetase (alias of GCAT) catalyzes the conversion of L-threonine to glycine [39].

**Table 1:** Top 20 protein-protein interactions from IDBOS ranking

	**Protein*i***			**Protein*j***		**Protein-protein interaction*i*-*j***			
**Symbol**	**Occurrence**	**Name**	**Symbol**	**Occurrence**	**Name**	**Occurrence** ***O***_***ij***_	**Z-score**	**Z-rank**	**Average rank**
**GCAT**	2	Glycine C-acetyltransferase	**TDH**	2	L-threonine dehydrogenase	2	577.4	11.0	1328.0
**CXCL16**	4	Inducible T cell co-stimulator	**CXCR6**	4	Inducible T cell co-stimulator ligand	4	565.7	21.0	1333.0
**CAPG (NCAPG)**	2	Non-SMC condensin I, G	**NCAPH**	2	Non-SMC condensin I, H	2	534.5	22.0	1333.5
**ICOS**	5	Inducible T cell co-stimulator	**ICOSLG**	5	Inducible T cell co-stimulator ligand	5	507.7	23.0	1334.0
**GPR103 (QRFPR)**	3	QRFP receptor	**P518(QRFP)**	3	Pyroglutamylated RFamide peptide	3	474.3	28.0	1337.0
**BCKDHA**	5	Branched chain keto acid dehydro. E1, alpha	**BCKDHB**	4	Branched chain keto acid dehydro. E1, beta	4	471.4	29.0	1337.5
**ARTN**	2	Artemin (GDNF family)	**GFRA3**	2	GDNF family receptor alpha 3	2	458.8	30.0	1338.0
**CX3CL1**	3	Chemokine (C-X3 -C motif) ligand 1	**CX3CR1**	3	Chemokine (C-X3-C motif) receptor 1	3	442.3	57.0	1351.5
**GIP**	7	Gastric inhibitory polypeptide	**GIPR**	9	Gastric inhibitory polypeptide receptor	7	431.7	58.0	1352.0
**POLG**	4	Polymerase (DNA directed), gamma	**POLG2**	3	Polymerase (DNA directed), gamma2	3	428.6	59.0	1352.5
**METTL1**	5	tRNA(m7G46)-methyltransferase	**WDR4**	2	tRNA (guanine-N(7)-)-methyltran. subunit WDR4	2	417.0	60.0	1353.0
**IL11**	3	Interleukin 11	**IL11RA**	2	Interleukin 11 receptor, alpha	2	408.2	61.0	1353.5
**HBA2**	68	Hemoglobin, alpha 2	**HBB**	76	Hemoglobin, beta	59	395.5	76.0	1361.0
**CLEC2D**	2	C-type lectin domain family 2, member D	**KLRB1**	3	Killer cell lectin-like receptor subfamily B, 1	2	392.2	77.5	1361.8
**LTC4S**	2	Leukotriene C4 synthase	**MGST1**	3	Microsomal glutathione S-transferase 1	2	392.2	77.5	1361.8
**CD97**	4	Leukocyte antigen CD97	**DAF**	2	CD55 antigen	2	384.9	79.5	1362.8
**D2LIC**	2	Dynein, cyto-plasmic 2, light intermed. chain 1	**DNCH2**	3	Dynein, cytoplasmic 2, heavy chain 1	2	384.9	79.5	1362.8
**IL22**	6	Interleukin 22	**IL22RA1**	4	Interleukin 22 receptor, alpha 1	4	383.1	81.0	1363.5
**CD200**	3	MRC OX-2 antigen	**CD200R1**	2	CD200 receptor 1	2	378.0	82.0	1364.0
**MLN**	4	Motilin	**MLNR**	7	Motilin receptor	4	369.8	98.0	1372.0

The top-20 ranked protein-protein interactions (PPIs) as identified by the interaction detection based on shuffling (IDBOS) method. We also provided the number of times each individual protein was observed in the aggregated data set, the number of times the PPI was observed, and the corresponding Z-score and associated fractional rank. All PPIs in this table have p-values <10^−6^, i.e., in the 10^6^ randomization shuffles each of these PPIs were never observed to occur more times than they were actually observed in the original data set. In the data set there were 5,288 PPIs with zero p-values and, hence, we assigned their uniform p-value rank to be 2,644.5 (5288/2). This value was used together with each Z-rank to create the average rank in the last column. The estimated Z-score of a PPI depends on the number of times each protein was observed in the original aggregated data set.

**Table 2:** Top 20 protein-protein interactions from occurrence ranking

	**Protein*i***			**Protein*j***		**Protein-protein interaction*i*-*j***			
**Symbol**	**Occurrence**	**Name**	**Symbol**	**Occurrence**	**Name**	**Occurrence** ***O***_***ij***_	**Z-score**	**Z-rank**	**Average rank**
**MDM2**	466	Mouse double minute 2 homolog	**TP53**	1097	Tumor protein p53	130	86.0	3355	2999.8
**TP53**	1097	Tumor protein p53	**UBC**	1899	Ubiquitin C	64	18.4	30486	16565.3
**HBA2**	68	Hemoglobin, alpha 2	**HBB**	76	Hemoglobin, beta	59	395.5	76	1360.3
**CBL**	414	Cas-Br-M ecotro- pic sequence	**EGFR**	626	Epidermal growth factor receptor	46	42.6	11054	6849.3
**CBL**	414	Cas-Br-M ecotro- pic sequence	**GRB2**	916	Growth factor receptor bound 2	44	33.2	15615	9129.8
**FANCA**	217	Fanconi anemia, complementation A	**FANCG**	143	Fanconi anemia, complementation G	43	117.3	1808	2226.3
**EGFR**	626	Epidermal growth factor receptor	**UBC**	1899	Ubiquitin C	41	15.8	34796	18720.3
**BRCA2**	199	Breast cancer 2, early onset	**RAD51**	171	DNA repair protein RAD51 homolog 1	40	104.4	2288	2466.3
**HIF1A**	207	Hypoxia inducible factor 1, alpha	**VHL**	351	von Hippel-Lindau tumor suppressor	39	69.2	5071	3857.8
**HRAS**	164	Ha-Ras1 proto-oncoprotein	**RAF1**	281	Proto-oncogene c-RAF	38	84.8	3444	3044.3
**SNAP25**	131	Synaptosomal-associated protein, 25kDa	**STX1A**	183	Syntaxin 1A	37	114.7	1920	2282.3
**MAX**	145	MYC associated factor X	**MYC**	382	Proto-oncogene c-Myc	36	73.4	4568	3606.3
**BARD1**	109	BRCA1 assoc. RING domain 1	**BRCA1**	448	Breast cancer 1, early onset	35	75.9	4279	3461.8
**GRB2**	916	Growth factor receptor bound 2	**SHC1**	317	SHC transforming protein 1	35	30.1	17593	10118.8
**CDH1**	167	Cadherin 1, type 1	**CTNNB1**	470	Catenin, beta 1	34	57.8	6876	4760.3
**E2F1**	168	E2F transcription factor 1	**RB1**	385	Retinoblastoma 1	34	64.0	5853	4248.8
**GRB2**	916	Growth factor receptor bound 2	**SOS1**	125	Son of sevenless homolog 1	32	44.9	10232	6438.3
**CCNA2**	109	Cyclin A2	**CDK2**	286	Cyclin-dependent kinase 2	31	84.3	3485	3064.8
**EGF**	76	Epidermal growth factor	**EGFR**	626	Epidermal growth factor receptor	31	68.2	5211	3927.8
**NFKBIA**	231	Nucl. factor of kappa light chain gene enhancer in B-cells	**RELA**	314	V-rel reticulo-endotheliosis viral oncogene homolog A	30	53.1	7932	5288.3

Top 20-ranked protein-protein interactions (PPIs), as identified by the number of times they were observed in the aggregated data set. The quantities and calculations are annotated as in Table 1, except that the data were sorted in decreasing order of PPI occurrence *O*_*ij*_. The proteins involved in these interactions are easily recognizable, and this partly reflects an underlying study-bias of focusing research efforts on a smaller set of high-interest proteins.

Not only were the top-ranked sets different, but the remaining bulk of the interactions also showed considerable changes in rank when the IDBOS p-values were non-zero (See Additional files 3 and 4). To evaluate the effect of this re-ranking of interactions, we then asked whether these rankings have any impact in retrieving biological information. We addressed this question by comparing the ability of the two schemes to identify known and inferred biological relationships based on rank. The hypothesis we tested was that higher-ranked subsets of the data sets are better at retrieving biological information, and that IDBOS ranking provides a more efficient way of retrieving this information than ranking solely based on frequency of occurrence.

### Evaluation of ranking schemes as measures of identifying interacting proteins

To assess different PPI ranking schemes, we constructed six benchmark reference sets derived from high-quality experimental studies as detailed in Materials and methods. These independent data sets comprise PPIs detected using *1*) far-Western blotting, *2*) isothermal titration calorimetry, *3*) nuclear magnetic resonance, *4*) surface plasmon resonance, *5*) direct interactions from protein complex structures from the PDB, and *6*) homologous human PPIs derived from actual mouse PPI data. These benchmark sets tested the ability of the ranked data to retrieve known interactions derived from a variety of experimentally determined PPI data sets.

Using these six benchmark reference sets, we compared the IDBOS-ranked data set with a set ranked by PPI frequency of occurrence, a set ranked using the hypergeometric test [23] (see Materials and methods, Additional file 5), and two random data sets. The random data sets included a set in which the PPIs themselves were retained, but their ranks were assigned randomly, and a set where both the interactions themselves were randomized and were assigned a random rank. In addition, we included the results obtained by directly using the nine individual data sources that formed the aggregated collection of human PPIs. The objective here was to assess each ranking method’s capability to retrieve true interactions in each of the reference sets, as a function of rank, i.e., higher-ranked data should contain a larger fraction of true interactions than lower-ranked data.

To make a fair comparison within each benchmark reference set, for each set we defined a protein pair from the aggregated data set as a judgeable interaction if both proteins appeared in the reference set; otherwise, we assumed that the pair could not be judged by the reference set. If a judgeable interaction was observed in the reference set, we termed it a true interaction. A good scheme would rank true interactions before other judgeable interactions. To quantify the ability to retrieve true interactions in each scored set, we extracted the judgeable subset and, using each corresponding rank as a threshold, we counted the numbers of judgeable and true interactions with scores above the threshold. We defined the number of true interactions as *coverage* and the fraction of true interactions among those judgeable interactions as *accuracy*.

**Figure 2:**
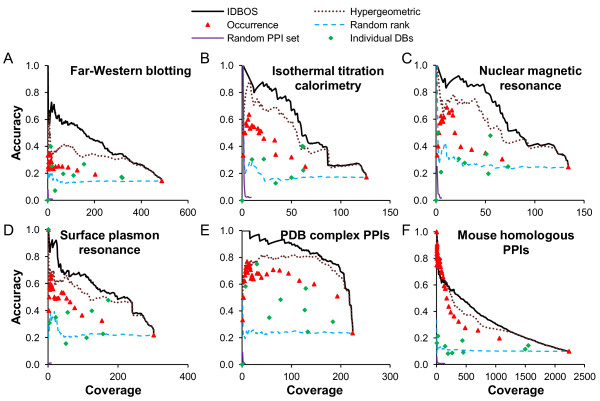
**Retrieving protein-protein interactions.** The proposed interaction detection based on shuffling (IDBOS) ranking scheme was compared with hypergeometric test ranking, frequency of occurrence ranking, one randomly ranked set (where the observed protein-protein interactions [PPIs] take random ranks), a randomly rewired interaction set, and nine individual data sets. We compared these ranking schemes using benchmark reference sets derived from six independent experimental data sets, namely: (**A**) far-Western blotting, (**B**) isothermal titration calorimetry, (**C**) nuclear magnetic resonance, (**D**) surface plasmon resonance, (**E**) protein pairs in direct contact with each other within protein complex structures collected from PDB (contacts determined using the jointly buried surface area), and (**F**) mouse homologous protein interactions. We assumed that each reference set could only judge a PPI when each of its two proteins actually appeared in this reference set (judgeable). We assessed performance by *coverage* (number of true interactions) and *accuracy* (fraction of true interactions among judgeable interactions) as a function of varying ranking thresholds (when applicable). The higher-ranked thresholds are to the left in the plots. In the set scored by occurrence alone, multiple PPIs possessed the same score, and the second right-most symbol entry corresponds to the collection of PPIs with an observed occurrence ≥2.

Figure 2 shows the results of this analysis for the six interaction benchmark reference sets. As expected, the completely random PPI data set had no capability to retrieve direct protein interaction data. For the network created by assigning random ranks to existing PPIs, the accuracy was almost uniform at any given coverage.

IDBOS, hypergeometric, and frequency of occurrence ranking were each associated with enhanced accuracy at higher ranks, thus indicating that true interactions ranked before other judgeable interactions. We assessed the overall performance of the ranking methods by calculating relative improvement compared to using no ranking, which is equivalent to assigning random ranks to the existing data. For the *accuracy* as a function of *coverage* plots shown in Figure 2, we calculated the average *accuracy*<A>over all ranks *r* as,

(3) $$<A>=\frac{1}{N}\sum_{r}accuracy(r)\times n(r)$$

where *n*(*r*) is the number of true PPIs at rank *r* and *N* is the sum of all *n*(*r*)’s. The gain of using IDBOS, hypergeometric, or occurrence as a ranking method was estimated by comparing the average *accuracy*<A>to the randomly ranked data $<A_{R}>$as,

(4) $$Gain=\left(\frac{<A>-<A_{R}>}{<A_{R}>}\right)\times 100$$

Table 3 lists the gains in average accuracies in each reference set for the different ranking schemes. The IDBOS ranking scheme shows the greatest increase compared to using randomly assigned ranks, achieves a two-fold increase in accuracy compared with using frequency of occurrence ranking, and consistently outperforms the hypergeometric ranking. In practice, one should also note that selecting any high-accuracy subset of PPIs based on ranked interaction data creates a smaller data set and lowers the overall PPI coverage.

**Table 3:** Evaluation of different ranking schemes

**Reference set**	**IDBOS**		**Hypergeometric**		**Occurrence**		**Random rank**
	<*A*>	*Gain*	<*A*>	*Gain*	<*A*>	*Gain*	<*A*>
Far-Western blotting	0.409	184%	0.313	118%	0.178	24%	0.144
Isothermal titration calorimetry	0.542	215%	0.469	173%	0.286	66%	0.172
Nuclear magnetic resonance	0.632	157%	0.534	117%	0.331	34%	0.246
Surface plasmon resonance	0.567	160%	0.496	128%	0.320	47%	0.218
PDB complex PPIs	0.842	258%	0.746	217%	0.566	141%	0.235
Mouse homologous PPIs	0.318	222%	0.293	198%	0.207	110%	0.099

The average accuracy < *A* > and gain associated with interaction detection based on shuffling (IDBOS), hypergeometric test ranking, and occurrence-ranking schemes were estimated using Equations 3 and 4. The value associated with the randomly ranked data set does not represent a random protein-protein interaction (PPI) data set as only the rank, and not the interactions themselves, were randomized. The tested reference sets contain PPIs derived from *1*) far-Western blotting, *2*) isothermal titration calorimetry, *3*) nuclear magnetic resonance, *4*) surface plasmon resonance experiments, *5*) known protein crystal complexes contained in the Protein Data Bank (PDB), and *6*) those derived from experimentally determined mouse PPIs. We tested whether the numerical differences in values of < *A* > for each pairwise comparison within each reference set were due to chance using the t-test, and rejected this hypothesis with a p-value of <10^-6^.

The independent scores (Z-scores or p-values) were typically better than, or equivalent to, the hypergeometric method: Z-score ranking was close to the IDBOS, and better than the hypergeometric, whereas the p-value ranking was not as good as the hypergeometric. For example, using the PDB benchmarking set, the gains of the IDBOS, Z-score, hypergeometric, and p-value methods relative to the average *accuracy* of the randomly ranked data were 258%, 253%, 217%, and 216%, respectively; using the mouse homologous benchmarking set, the gains were 222%, 211%, 198%, and 155%, respectively. Thus, the IDBOS combined rankings consistently outperformed all attempted scoring schemes.

### IDBOS versus hypergeometric test rankings

The observed differences in Table 3 and Figure 2 between the IDBOS and hypergeometric test rankings warrants further comment. The fundamental difference between the IDBOS and hypergeometric test rankings lies in how they account for interactions between self-interacting proteins. For the IDBOS randomized data, each pair of proteins was re-assigned an interaction partner such that no self-interacting protein pairs remained in the final set used to construct the probability density distribution functions. In the hypergeometric test, self-interacting protein pairs were assigned finite probabilities of occurrence based on a background distribution for each protein pair, which was different from IDBOS. Conceptually, the sum of the probability of observing all interactions with a given protein A among all other proteins p_AA_, p_AB_, p_AC_, etc., was the same in both schemes. However, the constraint that p_AA_ was zero in IDBOS and p_AA_ was non-zero in the hypergeometric test, re-distributed the probabilities such that any interaction probability p_AB_ between protein A and another protein B could be different in the two schemes. This strongly influenced the probability of detecting proteins that occurred with high frequency in the data set.

**Figure 3:**
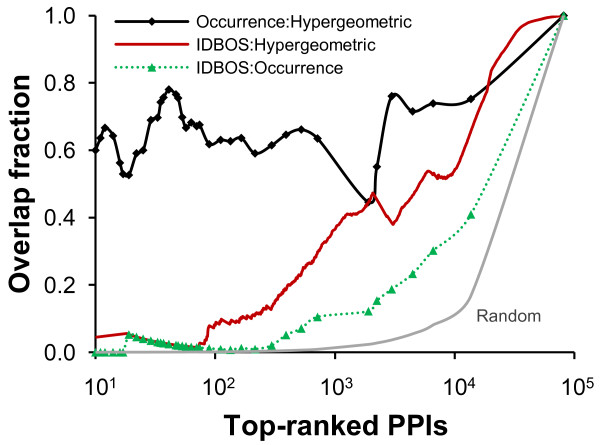
**Overlap of top-ranked protein interactions.** The overlap of top-ranked protein-protein interactions (PPIs), based on a pairwise comparison of three interaction evaluation schemes: interaction detection based on shuffling (IDBOS), hypergeometric test ranking, and frequency of occurrence ranking. The overlap between hypergeometric and occurrence rankings was considerable. The right-most point in the graph corresponds to the case where all interactions are included and, by definition, the three schemes overlap. For intermediate rankings, IDBOS shows considerably lower overlap with either method, indicative of distinct and different sets of top-ranked PPIs. The right-most curve (Random) shows the expected overlap fraction of top-ranking PPIs from two completely random rankings, emphasizing that even though the overlap fraction between IDBOS and Occurrence ranking was low, it was considerably higher than what would be expected by chance alone.

The effect of including or excluding self-interacting protein pairs was magnified in the evaluation of interactions involving popular proteins. In the hypergeometric test, the likelihood of randomly generating self-interacting protein pairs is roughly proportional to the square of the number of times the protein appears in the data set. In practice, this leads to an underestimate of the occurrence of non-identity pairs in the random data, and this increases the significance attached to the observed non-identity PPIs involving popular proteins. The effect on ranking PPIs was considerable, e.g., among the top 20 occurrence-ranked interactions listed in Table 2, 11 appeared in the top 20 hypergeometric test ranking scheme. In fact, the first occurrence-ranked interaction between MDM2 and TP53 was still the second ranked interaction based on the hypergeometric test. Figure 3 shows the overlap of interactions among top-ranked PPIs based on IDBOS, the hypergeometric test, and frequency of occurrence rankings. As exemplified above, there was a noteworthy overlap (>0.40) between the ranking results in the frequency of occurrence and the hypergeometric test rankings for all ranks tested. In contrast, the first 10^3^ top-ranked IDBOS PPIs showed low overlap (~0.10) with the frequency of occurrence ranked PPIs, but higher similarity (~0.36) to the ranked PPIs identified using the hypergeometric test. Thus, the high overlap between occurrence and hypergeometric rankings suggested that the inadvertent biases introduced by frequent investigations of popular proteins could not be completely disentangled by the hypergeometric-ranking scheme. Furthermore, we verified that consensus ranking schemes that included the average rank of all three ranking methods, or combining IDBOS and hypergeometric test rankings only, did not increase the accuracy beyond using IDBOS rankings (data not shown). Instead, we contend that the corresponding probability density distribution functions generated by IDBOS are the most appropriate to gauge unbiased PPIs in this data set.

### Enrichment of known domain-domain interactions

**Figure 4:**
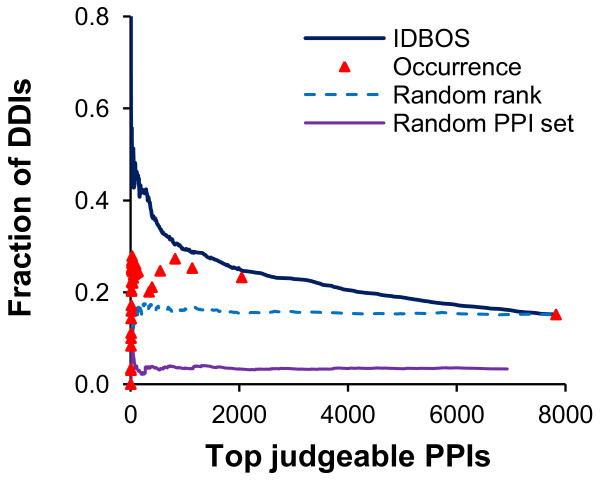
**Recovery of known domain interactions from protein interactions.** The proposed interaction detection based on shuffling (IDBOS) ranking scheme was compared with the frequency of occurrence ranking, one randomly ranked set (where the observed protein-protein interactions (PPIs) take random ranks), and a randomly rewired interaction set in identifying known domain-domain interactions (DDIs). We assessed performance by calculating the fraction of true DDIs among the judgeable interactions as a function of varying ranking thresholds. In the set scored by occurrence alone, multiple PPIs possessed the same score, and the second right-most symbol corresponds to the collection of PPIs with an observed occurrence ≥2.

The hypothesis that PPIs are mediated by a smaller set of specific domain-domain interactions (DDIs) that are repeatedly used, can be exploited by inferring DDIs from known PPIs and then predicting novel PPIs from the inferred DDI set [40,41]. A high-confidence PPI set, such as the collection of highly ranked PPIs, is more likely to contain reliable DDIs. To confirm this, we investigated the fraction of known DDIs among all candidate domain pairs as a function of the rank threshold. As before, we only considered judgeable protein pairs whose domains appear in the set of known DDIs. The known DDI set was inferred from the PDB crystal structures [42]. As two random controls, we also randomly ranked and randomly rewired the judgeable protein pairs. Figure 4 shows that more stringent ranking thresholds (represented by fewer “Top judgeable PPIs” in Figure 4) resulted in higher enrichments of DDIs among all domain pairs between interacting proteins for both the IDBOS and frequency of occurrence rankings. This behavior was in contrast with the two random controls, which did not show such enrichments. Table 4 quantifies the improvement gained by ranking the data relative to no rankings. Within the same top number of PPIs, the IDBOS set had a higher enrichment of known DDIs than sets ranked either by the hypergeometric test or by frequency of occurrence, indicating a potential application of the IDBOS-ranked PPI sets for the inference of accurate DDIs.

**Table 4:** Domain-domain interaction enrichment

**Reference set**	**IDBOS**		**Hypergeometric**			**Occurrence**		**Random rank**
	*Fraction*	*Gain*	Fraction	*Gain*	*Fraction*	*Gain*	*Fraction*
DDI	0.225	48%	0.215	42%	0.174	15%	0.152

The fraction of true domain-domain interactions (DDIs) retrieved with interaction detection based on shuffling (IDBOS), hypergeometric test ranking, occurrence ranking, and randomly ranked data. We calculated the reported fraction and gain associated with the different ranking schemes as outlined in Equations 3 and 4. We tested whether the numerical differences in the fraction values for each pairwise comparison were due to chance using the t-test, and rejected this hypothesis with a p-value of <10^-24^.

### Evaluation of ranking PPIs as a means of retrieving biological information

Up until now, the different scoring schemes were used to evaluate reference data sets that can be considered to be directly related to interacting proteins. We next evaluated the improvement that could be gained by using the differently ranked data sets to rank-order the interactions in reference data sets that are presumed to be enriched with interacting proteins, i.e., in sets of proteins that share the same Kyoto Encyclopedia of Genes and Genomes (KEGG) pathway, are implicated in the same disease, share Gene Ontology (GO) function, or tissue mRNA expression levels.

### Case I: Enrichment of KEGG co-pathway gene pairs

**Figure 5:**
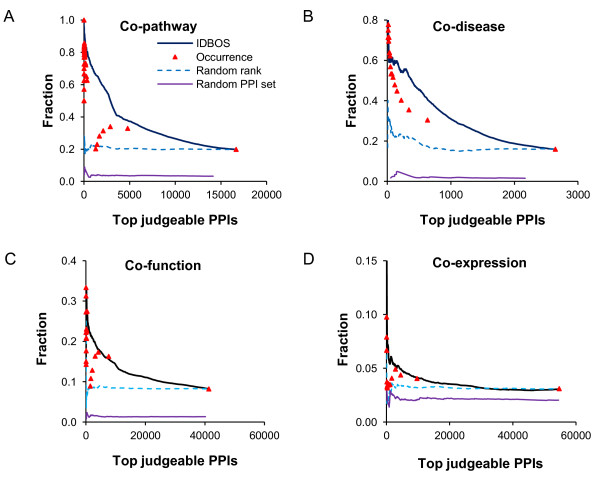
**Recovery of biological co-annotations from protein interactions.** The proposed interaction detection based on shuffling (IDBOS) ranking scheme was compared with the frequency of occurrence ranking, one randomly ranked set (where the observed protein-protein interactions (PPIs) take random ranks), and a randomly rewired interaction set in retrieving biological relationships from diverse reference sets. The reference sets were: (**A**) co-pathway gene pairs, (**B**) co-disease susceptibility gene pairs, (**C**) functionally related gene pairs, and (**D**) tissue co-expressed gene pairs. We assessed performance by calculating the enrichment fraction of true PPIs among the judgeable interactions as a function of varying ranking thresholds. In the set scored by occurrence alone, multiple PPIs possessed the same score, and the second right-most symbol corresponds to the collection of PPIs with an observed occurrence ≥2.

Genes encoding interacting proteins are more likely to be part of the same pathway. Hence, the fraction of PPIs that are annotated to belong to the same pathway should be larger in sets containing higher-ranked PPIs. We used KEGG pathway data [43] to define co-pathway gene pairs, as outlined in the Materials and methods. For each investigated PPI set, we excluded protein pairs involving proteins that did not participate in any KEGG pathway (these were deemed to be non-judgeable). Figure 5A shows the results of this analysis. We confirmed the correlation between co-pathway membership of a protein pair and its rank threshold in both the IDBOS and the frequency of occurrence ranking schemes, as well as the absence of correlation in the two random controls. Overall, IDBOS ranking outperformed frequency of occurrence ranking by a factor of four for enrichment of co-pathway gene pairs among selected top-ranking PPIs (Table 5), and the gain seen in hypergeometric ranking was substantially smaller than that of IDBOS ranking. There is a discontinuity in the curve for the frequency of occurrence ranking scheme, which was caused by hundreds of interactions between three subunits of G protein (GNAL, GNB1, and GNGT1) and olfactory proteins which were supported by seven publications (Additional file 1). We reviewed these publications and found that this literature support was weak, with some based solely on gene expression evidence of those olfactory genes [44]. Among these hundreds of PPIs, those involved with GNB1 (beta subunit) or GNGT1 (gamma subunit) were not supported by KEGG pathway data, resulting in a sharp drop in accuracy.

**Table 5:** Evaluation of ranked interactions to detect biological relationships

**Reference set**	**IDBOS**		**Hypergeometric**		**Occurrence**		**Random rank**
	*Fraction*	*Gain*	Fraction	*Gain*	*Fraction*	*Gain*	*Fraction*
Co-pathway	0.350	76%	0.315	59%	0.235	18%	0.199
Co-disease	0.306	91%	0.298	86%	0.214	34%	0.160
Co-function	0.122	48%	0.123	50%	0.095	16%	0.082
Co-expression	0.036	15%	0.035	14%	0.033	6%	0.031

The fraction of biological associations retrieved using interaction detection based on shuffling (IDBOS), hypergeometric test ranking, occurrence ranking schemes, and randomly ranked data. We calculated the reported fraction and gain associated with the different ranking schemes as outlined in Equations 3 and 4. The four reference sets tested contain putative biological relationships pertaining to protein interactions that are in the same Kyoto Encyclopedia of Genes and Genomes (KEGG) pathway (co-pathway), implicated in the same disease (co-disease), share Gene Ontology (GO) function (co-function), or tissue expression levels (co-expression). We tested whether the numerical differences in the fraction values for each pairwise comparison within each references set were due to chance using the t-test, and rejected this hypothesis with a p-value of <10^-2^.

### Case II: Enrichment of co-disease gene pairs

Genomic variations may underlie different susceptibilities to disease, e.g., individuals with specific single nucleotide polymorphisms (SNPs) can develop, or be pre-disposed to, a particular disease phenotype. Furthermore, genes encoding interacting proteins are more likely to occur within the same disease classification [45]. Using the gene co-disease data extracted by Goh et al. [45] from the Online Mendelian Inheritance in Man (OMIM) dataset [46], we investigated the enrichment of co-disease gene pairs as a function of rank threshold (Figure 5B, Table 5). A gene pair was termed a co-disease gene pair if their SNPs led to susceptibility to the same disease. With this co-disease gene pair set, we also extracted judgeable protein pairs whose encoding genes appeared in this co-disease set (i.e., genes that have an actual disease annotation). Similar to the previous analyses, the IDBOS-scored set showed an enhanced probability of identifying existing disease-susceptible genes as compared with the frequency of occurrence ranking scheme, hypergeometric-based rankings, or random controls.

### Case III: Enrichment of GO co-function gene pairs

Figure 5C shows the ability of the IDBOS and frequency of occurrence ranking schemes to identify PPIs whose proteins share the same gene ontology (GO) function. Both ranking schemes displayed a similar rank-dependent enrichment of co-function proteins compared to the randomly ranked (all ranks have the same importance) and randomly rewired (interactions are random) data set. Table 5 indicates that the improvements of the ranking schemes compared to randomly ranked data are relatively modest in retrieving functionally related gene pairs. As pointed out by Gillis and Pavlidis [28], the ability of PPI networks to distinguish co-functionality is largely influenced by the multifunctional nature of the gene annotations schemes themselves. In this case, the IDBOS, hypergeometric, and occurrence ranking schemes themselves will also have less influence on the retrieval of functionally related protein pairs compared to the annotation scheme itself.

### Case IV: Enrichment of tissue co-expression gene pairs

Finally, we examined the occurrence of interacting protein pairs in a large data set that maps out the global mRNA expression levels for ~20,000 human genes in 32 normal tissues [47]. For detail on the construction of this reference set, see Materials and methods. The biological hypothesis tested here examined co-expression of two mRNAs in the same tissue as indicative that the corresponding proteins had higher probability of interaction. Figure 5D shows the fraction of PPIs retrieved as a function of ranking scheme. Overall, the fraction of PPIs contained in the co-expression data set was lower than that of the other biological reference sets examined above. However, the investigated ranking schemes were able to produce ranked data sets that contain a slightly larger fraction of co-expressed PPIs than using no ranking (Table 5).

### Topological differences of ranked PPI networks

**Figure 6:**
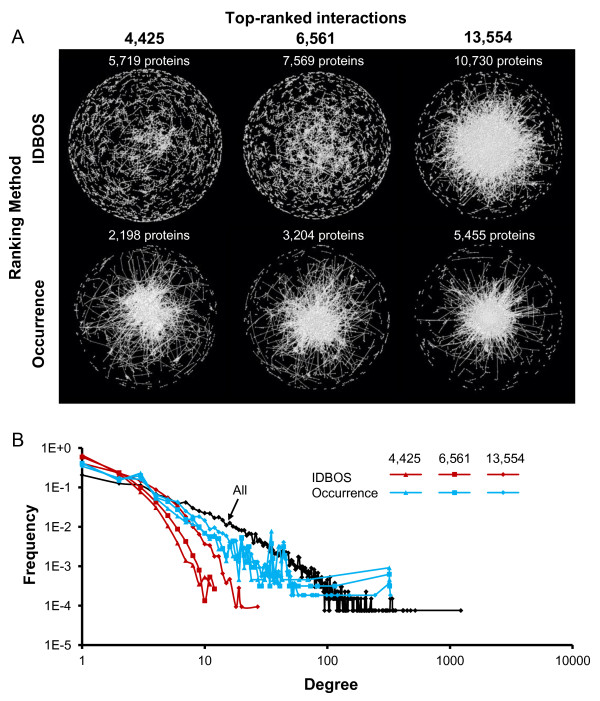
**Network topology of ranked interactions**. (**A**) We reconstructed the corresponding top-ranked protein-protein interactions (PPIs) networks using both interaction detection based on shuffling (IDBOS) ranking and frequency of occurrence ranking. The columns of 4,425, 6,561, and 13,554 top-ranked interactions corresponds to selecting PPIs with ≥ 4, ≥ 3, and ≥ 2 reported occurrences, respectively. These interactions were distributed among roughly twice as many proteins using IDBOS ranking than in occurrence ranking. (**B**) The degree distribution for each selected PPI network was analyzed and compared to the distribution of all aggregated interactions (All). The higher-ranked PPI data sets were associated with fewer proteins that had a large number of interacting partners (hubs). As visualized in (**A**), the top-ranked networks were instead characterized by more evenly distributed interactions in a loosely interconnected network.

Data sets of protein interactions are commonly represented as graphs or networks of protein interactions, and applying network topology metrics to characterize the biological role of PPIs has attracted wide attention [9,10,22,48]. The presence of confounding factors, e.g., protein abundance [49] or popularity [12], can strongly influence the topological properties of the network. Here, we used subsets of highly ranked PPIs derived from using either IDBOS or occurrence ranking to select smaller high-confidence networks. Figure 6A shows the overall networks constructed from the top-ranking PPIs corresponding to 4,425, 6,561, and 13,554 interactions selected based on the number of PPIs that have more than or equal to four, three, and two reported occurrences, respectively. Figure 6B shows the corresponding degree distribution, i.e., the distribution of the number of unique interacting partners each protein has (Degree), as well as the overall degree distribution for the entire network (All).

Selecting highly ranked subsets of PPIs, using either IDBOS or occurrence, had the effect of reducing the number of high-degree proteins (hubs) in the overall network. This effect was most pronounced in the top-ranked IDBOS networks, which effectively down-weights interactions of popular proteins. The resulting top-ranked IDBOS networks were not dominated by hub interactions, but show a more distributed interaction network, with distinct topological and scaling properties.

## Discussion

Single experiments designed for large-scale detection of PPIs judge multiple observations of the same PPI as evidence for the confidence of the interaction. Multiple observations, across many different experiments, of a particular PPI also lend confidence that this interaction occurs in nature. One would then conclude that if one aggregated all known PPI experiments, the more times a particular PPI occurred, the more confident one would be of the interaction. We found a somewhat counterintuitive result in that ranking PPIs in order of the number of times a PPI has been observed was actually not the optimal way of assessing the importance of frequently reported interactions. Instead, we used the IDBOS method, which ranks interacting protein pairs in the observed PPI data sets as compared to random occurrences derived from an empirically reconstructed probability density distribution function specific to each interaction. We then used these ranks to order the PPI data and showed that this approach consistently identified more known protein associations in numerous benchmark reference sets than using either hypergeometric test rankings or simply frequency of occurrence. The improvement of IDBOS over the hypergeometric test results stems from the differences in the underlying random distributions in the two schemes. While the hypergeometric test does not impose any limitations on self-interacting proteins, by construction, the IDBOS procedure sets their probability of occurrence to zero even for the random case. The input data contain no self-interacting proteins; therefore, the corresponding random probability density distribution functions generated by IDBOS seem to be the most appropriate to gauge unbiased PPIs in this data set.

We evaluated the improvement in using ranked PPI data to identify known protein relationships in eleven independent reference sets, including direct and indirect readouts of protein interactions. We used a large number of different reference sets to perform as comprehensive an analysis as possible, as each reference set only covers a specific subset of protein interactions. It was clear that ranking interactions was of value, because higher-ranked PPIs had better success at retrieving protein associations. Across all sets investigated, occurrence ranking improved the performance by 46% compared to using non-ranked data, hypergeometric ranking achieved a 109% increase, and the IDBOS ranking scheme achieved a 134% increase. This conformed to the assumption that aggregating and creating high-confidence subsets of data creates added value, albeit at a cost of reducing the overall number of PPIs that can be considered.

## Conclusions

We have developed a statistical approach to infer subsets of high-confidence human PPIs, and showed that ranked data can consistently enrich the accuracy of the retrieved PPI data in these sets. Our IDBOS method was more successful in ranking interactions than using either the number of times an interaction has been observed across experiments or rankings based on the hypergeometric test. Furthermore, using either IDBOS or hypergeometric scoring schemes generates unique ranks for almost all interactions, as opposed to the frequency of occurrence method, in which many interactions have the same integer score corresponding to the number of observed occurrences. The IDBOS ranking increased accuracy and enrichment of protein interaction data associated with PPIs by more than two-fold compared to simply ranking interactions based on observed occurrences. We achieved this improvement by comparing the observed interaction data with a probability density distribution function that does not inflate the statistical importance of interactions associated with frequently studied proteins. These results suggest that using our high-confidence protein interactions at different levels of confidence could help clarify the dependence on confidence on topological and biological properties associated with human protein networks.

## Materials and methods

### Statistical scoring of PPIs from aggregated experimental data

We downloaded the collection of PPIs in October 2011 from the nine databases that covers the bulk of all known experimentally determined PPIs. Databases of non-primary nature, i.e., containing aggregated data and/or predicted and inferred interactions, were excluded. From this collection, we extracted the subset of human physical PPIs, consisting of PPIs from both large-, semi-large-, and small-scale experimental studies. Because Reactome has no standardized annotations describing physical associations or direct interactions, instead we extracted PPIs annotated as “direct complexes.” We treated each interaction reported by each study (identified by a unique publication ID) as a unique record by deleting redundant copies arising from the overlaps among the nine databases. In total, we analyzed 13,369 proteins, 80,980 PPIs, and 116,134 records (Additional file 1). The computational procedure to generate 10^6^ random realizations of the PPI data set and compute the corresponding p-values and Z-scores took ~2,000 minutes on a dual core Xeon Irwindale 3.6 GHz 64-bit Linux server equipped with 4 GB of RAM. We used fractional rankings, i.e., PPIs that had the same score received the same ranking number, which is the mean of what they would have received under ordinal rankings. The Supplementary material provides the scored and ranked PPI data set, with equivalently ranked interactions tabulated in arbitrary order.

To rank interactions based on an alternative statistical method compared to IDBOS and frequency of occurrence, we included ranks based on a hypergeometric test [23]. For two proteins, *i* and *j*, given that the interaction between *i* and *j* occurred *N*_*ij*_ times, the probability (*p*_*ij*_) for these two proteins to have this or a larger number of interactions by chance was approximated using a hypergeometric distribution as follows:

(5) $$p_{\mathrm{ij}}=\sum_{n=N_{\mathrm{ij}}}^{\min(N_{i},N_{j})}\;\left(\begin{array}{c} N_{j}\\ n \end{array}\right)\left(\begin{array}{c} 2N-N_{j}\\ N_{i}-n \end{array}\right)/\left(\begin{array}{c} 2N\\ N_{i} \end{array}\right)\;$$

where *N*_*i*_ (*N*_*j*_) is the number of times protein *i* (*j*) was observed in the aggregated data set, and *N* is the total number of interactions in the aggregated data set of PPIs. This formulation is correct in the limit of *N* > > *N*_*i*_*N*_*j*_, which was satisfied in this data set. Finally, we ranked the PPI data based on the calculated probabilities.

### PDB interactions

We mapped all PDB sequences (downloaded in October 2010) to the human protein sequences stored in the UniProtKB/Swiss-Prot database (downloaded in June 2008) and obtained 649 protein complexes that contained at least two different human proteins. We then calculated the contact area of each intra-complex human protein pair using the program EMPIRE [50], to determine which protein pairs interact. For protein pairs occurring in multiple complexes, we selected the pair with the largest contact area as an interacting pair. We collected 281 direct protein interactions between 563 proteins, with contact areas ranging from 0.1 to 183 nm^2^, as a reference set for known human PPIs derived from structural data (Additional file 2).

### Reference sets from the HIPPIE database

We extracted four reference sets from the Human Integrated Protein-Protein Interaction rEferenc (HIPPIE) data set (http://cbdm.mdc-berlin.de/tools/hippie/ information.php, January 2012), each of which consists of PPIs detected by a top-scored experiment technique (score = 10, specified with European Bioinformatics Institute (EBI) term ID, and number of PPIs >100). The extracted data set contained PPIs derived from *1*) far-Western blotting, *2*) isothermal titration calorimetry, *3*) nuclear magnetic resonance, and *4*) surface plasmon resonance experiments.

### Homologous human PPIs from mouse data

We extracted the subset of mouse physical PPIs from the above collection and constructed a homologous human PPI set according to the sequence homology between mouse and human proteins defined by the National Center for Biotechnology Information (NCBI) homology mapping scheme (ftp://ftp.ncbi.nih.gov/pub/HomoloGene/, October 2009). We used this homologous human PPI set (containing 3,148 interactions between 5,632 proteins) as a reference data set reflective of direct protein interactions.

### Domain annotation and interaction data

We used the Pfam-A families of the Pfam21.0 database as the source for domain annotation [51]. We also downloaded the DDI set inferred from PDB crystal structures from the iPfam database [42]. This DDI set contains 4,030 interactions between 2,837 Pfam-A domains.

### Co-disease susceptibility gene pairs

We used the disease susceptibility gene data of Goh et al. [45], which was constructed by processing OMIM raw data [46]. There were 43,249 pairs between 3,670 genes whose two genes were susceptible to at least one common disease. We used these pairs to represent the co-disease susceptibility gene pairs.

### Co-function gene pairs

We downloaded the GO annotation data (http://www.geneontology.org, June 2010) to identify a set of functionally related gene pairs as a reference set. For each gene, we expanded its GO annotation list by including all ancestors of each member in the list. For two genes, *g* and *h*, given that they had *n*_*gh*_ GO annotations in common, the probability (*p*_*gh*_) for these two genes to have this or larger annotation overlap by chance was estimated from the hypergeometric distribution as follows:

(6) $$p_{\mathrm{gh}}=\sum_{i=n_{\mathrm{gh}}}^{\min(N_{g},N_{h})}\;\left(\begin{array}{c} N_{h}\\ n_{\mathrm{gh}} \end{array}\right)\left(\begin{array}{c} T-N_{h}\\ N_{g}-n_{\mathrm{gh}} \end{array}\right)/\left(\begin{array}{c} T\\ N_{g} \end{array}\right)\;$$

where *N*_*g*_ (*N*_*h*_) is the number of GO annotations of gene *g* (*h*) and *T* is the total number of unique GO annotations. We used –log(*p*_*gh*_) as the score and chose the top 1% of all gene pairs as a reference set, resulting in 1,502,420 co-function pairs for this reference set.

### Co-pathway gene pairs

The KEGG pathway data file (hsa_gene_map.tab) was downloaded from its Web site (ftp://ftp.genome.jp/pub/kegg/pathway/, June 2010), which lists the pathways that each annotated gene participates in. Similar to the co-function score described above, the co-pathway score was also computed using the hypergeometric distribution method. We chose the top 1% as the reference set of significant co-pathway gene pairs (139,841 pairs).

### Tissue co-expression of gene pairs

We used a global expression data set containing mRNA expression levels for ~20,000 human genes in 32 normal tissues derived from massively parallel signature sequencing [47]. The raw data set documents the abundance values of each short sequence signature tag (with a total of 182,727 tags) in 32 tissues. We mapped these tags to the regions of the human genome (hg18) that encode genes, in both orientations. We obtained 105,512 hits in the gene regions in both orientations and, among them, 68,855 hits in the gene orientation (p-value < 10^−2,000^), indicating that the tags were able to distinguish the transcribed orientation from the non-transcribed orientation in the genome. Assigning the tags that hit a gene region and orientation to the corresponding gene, we obtained a set of tags for each gene, resulting in a total of 14,516 genes having non-empty tag sets. We summed up the abundance tissue profiles of the tags of a gene to create its raw expression profile.

Furthermore, we computed the statistical significance of a gene being preferentially expressed in a tissue (termed “tissue specificity”) using an approach similar to that of Yu *et al.*[52], which identified tissue-specific genes from the NCBI Expressed Sequence Tag database. Let *e*_*k*_(*g*) be the expression level of gene *g* in tissue *k*. The total expression of gene *g* in all 32 tissues is,

(7) $$E(g)=\sum_{k}e_{k}(g)$$

and the expected total expression of all genes in tissue *k* is,

(8) $$E_{k}=\sum_{g}e_{k}(g)$$

If we randomly throw *E*(*g*) darts into 32 areas of sizes *E*_*k*_, *k* = 1, 2, …32, and each dart has a probability,

(9) $$q_{k}=\frac{E_{k}}{\sum_{m}E_{m}}$$

of hitting area *k*, we would expect to see *E*(*g*) *q*_*k*_ darts in this area with variance *E*(*g*)*q*_*k*_(*1-q*_*k*_). Similarly, if gene *g* were equally expressed across all tissues, the expected expression level in tissue *i* would be *E*(*g*)*q*_*k*_ with variance *E*(*g*)*q*_*k*_(*1-q*_*k*_). Thus, we used the corresponding *Z*-score,

(10) $$Z_{k}(g)=\frac{e_{k}(g)-E(g)q_{k}}{\sqrt{E(g)q_{k}(1-q_{k})}}$$

as the tissue specificity of gene *g* in tissue *k*. Accordingly, we defined the tissue co-expression score of genes *g* and *h* as,

(11) $$C_{\mathrm{gh}}=\sum_{m}Z_{m}(g)Z_{m}(h)$$

We chose the top 1% of these scored gene pairs as the reference set of co-expressed gene pairs to evaluate the PPI scoring approaches. There were 1,053,529 co-expression pairs in this set.

## Competing Interests

The authors declare that they have no competing interests.

## Authors’ contributions

XY, AW, and JR conceived of the study and participated in its design and coordination. XY collected the data, developed the algorithms, and performed the calculations. AW and XY drafted the original manuscript, which was edited by JR. All authors read and approved the final manuscript.

## Supplementary Material

Additional file 1 Aggregated human physical PPI data. This tab-delimited text file contains the aggregated PPI data based on publications collected from nine databases: BIND, BioGRID, DIP, HPRD, IntAct, MINT, MIPS, PDZBase, and Reactome. Click here for file

Additional file 2 Direct interactions supported by known complexes in PDB. This tab-delimited file contains the protein pairs that have direct contact in at least one protein complex in PDB. Click here for file

Additional file 3 IDBOS Ranked PPI data. This tab-delimited file contains the aggregated PPI data set with the corresponding IDBOS ranking. Click here for file

Additional file 4 Occurrence Ranked PPI data. This tab-delimited file contains the aggregated PPI data set with the corresponding occurrence ranking. Click here for file

Additional file 5 Hypergeometric Ranked PPI data. This tab-delimited file contains the aggregated PPI data set with the corresponding hypergeometric ranking. Click here for file
